# Expression de NF-κB, EGFR et HER3 dans l’adénocarcinome prostatique résistant à la castration: étude clinique et moléculaire chez 88 patients marocains

**DOI:** 10.11604/pamj.2026.53.144.51276

**Published:** 2026-03-26

**Authors:** Ilham Sadaoui, Imane Sadaoui, Hanaa Amrani Hassani Joutei, Hakima Benomar, Sellama Nadifi

**Affiliations:** 1 Laboratoire de Biologie Cellulaire et Génétique Moléculaire (LBCGM), Faculté des Sciences, Université Ibn Zohr, Agadir, Maroc,; 2 Laboratoire d'Anatomie et Cytologie Pathologiques de l'Institut Pasteur du Maroc, Casablanca, Maroc,; 3 Laboratoire de Génétique Médicale, Faculté de Médecine et de Pharmacie, Casablanca, Maroc,; 4 Département des Sciences Informatiques, Université Al Akhawayn, Ifrane, Maroc,; 5 Département de Neuroscience, Maladies Infectieuses et Substances Naturelles, Faculté des Sciences et Techniques, Mohammedia, Maroc

**Keywords:** Cancer de la prostate résistant à la castration, facteur de transcription NF-κB, récepteur HER3, Castration-resistant prostate cancer, NF-κB transcription factor, HER3 receptor

## Abstract

La progression de l'adénocarcinome prostatique (CaP) vers la résistance à la castration implique des dérèglements moléculaires complexes. Cette étude évalue l'implication de NF-κB et des récepteurs à l'EGF (EGFR, HER3) dans l'agressivité tumorale chez des patients marocains. Une étude rétrospective avec analyse immunohistochimique a été réalisée sur les tissus de 88 patients atteints de CaP. L'expression des sous-unités de NF-κB (p65, p50, RelB), de l'EGFR et de HER3 a été corrélée au statut de résistance à la castration et au score de Gleason (tests du Chi-carré et régression logistique). Une forte localisation nucléaire de NF-κB a été observée dans les cellules cancéreuses résistantes à la castration (p = 0,0005), constituant un marqueur prédictif de progression. Parallèlement, une surexpression de l'EGFR a été identifiée chez les patients hormono-réfractaires (OR = 5,37; p = 0,0010). L'expression nucléaire de HER3 était significativement corrélée à un grade de Gleason élevé (≥ 7) dans les tumeurs résistantes (p = 0,0064). L'activation nucléaire de NF-κB et de HER3, ainsi que la surexpression de l'EGFR, jouent un rôle clé dans l'évolution vers l'échappement hormonal. Ces molécules représentent des cibles thérapeutiques et des biomarqueurs pronostiques prometteurs pour la prise en charge du CaP à haut risque au Maroc.

## Abstract (en)

The progression of prostate adenocarcinoma (PCa) toward castration resistance involves complex molecular pathways. This study evaluates the role of NF-κB and EGF receptors (EGFR, HER3) in tumor aggressiveness among Moroccan patients. A retrospective study with immunohistochemical analysis of NF-κB subunits (p65, p50, RelB), EGFR, and HER3 was performed on tissue samples from 88 PCa patients. Results were correlated with castration resistance status and Gleason scores (using Chi-square tests and logistic regression). Strong nuclear localization of NF-κB was detected in castration-resistant cancer cells (p = 0.0005), serving as a predictive marker for progression. Simultaneously, high EGFR expression was identified in hormone-refractory patients (OR = 5.37; p = 0.0010). Nuclear HER3 expression significantly correlated with high Gleason grades (≥ 7) in resistant tumors (p = 0.0064). Nuclear activation of NF-κB and HER3, along with EGFR overexpression, plays a critical role in hormonal escape. These molecules represent promising therapeutic targets and prognostic biomarkers for high-risk PCa management in Morocco.

## Introduction

Au Maroc, le cancer de la prostate (CaP) représente un enjeu de santé publique majeur, se classant au deuxième rang des cancers masculins, derrière le cancer du poumon. Selon les dernières estimations du *Global Cancer Observatory* (GLOBOCAN 2022), le pays enregistre désormais environ 4 935 nouveaux cas annuels, soit 16,1% de l'ensemble des cancers masculins [1]. Il constitue la deuxième cause de mortalité par cancer chez l'homme au Maroc, avec plus de 2 030 décès par an. Les données du Registre des Cancers de la Région du Grand Casablanca confirment une incidence standardisée élevée, due au vieillissement de la population et à une détection accrue [2]. Environ 7% de la population masculine marocaine se situe dans la tranche d'âge à risque, avec une proportion non négligeable progressant vers un stade métastatique et hormono-réfractaire [3,4].

La résistance à la castration se caractérise par une réactivation de la signalisation du récepteur aux androgènes (RA) malgré le traitement anti-hormonal. Parmi les mécanismes moléculaires impliqués, l'activation du RA par des récepteurs aux facteurs de croissance, notamment l'EGFR *(Epidermal Growth Factor Receptor)* et HER3, joue un rôle prépondérant dans l'échappement thérapeutique [5]. À ce stade avancé, les options thérapeutiques demeurent principalement palliatives, ce qui explique le taux de mortalité élevé. L'estimation du risque de progression par les paramètres cliniques actuels étant limitée, l'identification de nouveaux biomarqueurs est cruciale pour combler cette lacune [6,7].

Le facteur de transcription NF-κB émerge comme un marqueur pronostique clé dans le développement du CaP. Une localisation nucléaire de la sous-unité transactivatrice RelA (p65) permet d'affiner le grade histologique pour mieux stratifier les patients à risque de progression agressive [8]. Par ailleurs, l'implication de la voie alternative de NF-κB a également été suggérée comme moteur de la résistance [9]. Dans ce contexte, notre étude vise, d'une part, à évaluer l'implication de l'expression de l'EGFR et de HER3 dans la réactivation du RA menant à l'hormono-indépendance chez des patients marocains. D'autre part, elle tend à clarifier le rôle de NF-κB en tant que facteur étroitement lié à la progression tumorale. Ces travaux pourraient contribuer à une meilleure gestion clinique et à l'élaboration de stratégies thérapeutiques ciblées pour les formes agressives de la maladie.

## Méthodes

**Patients et échantillonnage:** cette étude transversale a porté sur une cohorte de 88 patients marocains diagnostiqués d'un adénocarcinome prostatique. La population a été recrutée de manière multicentrique auprès de l'Institut Pasteur du Maroc et de plusieurs centres d'Anatomie et Cytologie Pathologiques (ACP) de Casablanca. Un échantillonnage exhaustif de tous les cas répondant aux critères d'inclusion a été réalisé entre janvier 2016 et décembre 2020, permettant d'identifier initialement 112 dossiers. Après exclusion des cas présentant un matériel insuffisant ou des données incomplètes, l'analyse finale a porté sur 88 patients, divisés en deux groupes: 58 cas de cancer de la prostate résistant à la castration (CPRC) et 30 cas hormono-sensibles (HS). Les échantillons tissulaires, constitués de pièces d'exérèse chirurgicale ou de biopsies fixées au formol tamponné à 10% et inclus en blocs de paraffine (FFPE), ont été collectés auprès de plusieurs centres d'anatomie pathologique à Casablanca. Les échantillons et les données cliniques ont été collectés sur une période de 5 ans, allant de janvier 2016 à décembre 2020. Pour chaque patient, une fiche clinique détaillée a été établie, incluant l'âge, le type de résection, la taille de la tumeur, le score de Gleason, le stade TNM et les symptômes cliniques. Le diagnostic a été confirmé par examen histologique au Laboratoire d'anatomie pathologique de l'Institut Pasteur de Casablanca. Cet examen a également permis de délimiter les zones tumorales d'intérêt pour la construction de matrices tissulaires (Tissue Microarrays - TMA). La taille de l'échantillon (N=88) a été déterminée par un échantillonnage exhaustif de tous les cas répondant aux critères d'inclusion durant la période d'étude mentionnée.

**Considérations éthiques:** l'étude a été menée conformément aux principes de la déclaration d'Helsinki. Un formulaire de consentement éclairé a été dûment signé par l'ensemble des participants. Le protocole a été validé par les autorités compétentes des centres de santé participants.

**Analyse immunohistochimique (IHC):** l'expression de six marqueurs moléculaires (RA, p65, p50, RelB, EGFR et HER3) a été analysée par immunohistochimie automatisée à l'aide du système Ventana Medical Systems Inc. (Tucson, AZ, USA). Les anticorps primaires ont été fournis par Santa Cruz Biotechnology (Santa Cruz, CA, USA). L'interprétation des lames a été réalisée par deux uropathologistes indépendants en aveugle des données cliniques. En cas de divergence, une troisième lecture a été effectuée. Le seuil de positivité pour le marquage nucléaire a été fixé à 1%. L'intensité du marquage a été évaluée selon une échelle semi-quantitative: i) 0: négatif; ii) 1+: intensité faible; iii) 2+: intensité moyenne; iv) 3+: intensité forte.

**Analyse statistique:** les cas présentant des blocs tissulaires inexploitables ou des fiches cliniques incomplètes ont été exclus de l'analyse afin de garantir l'intégrité des données. Le traitement statistique a été réalisé avec le logiciel Epi Info™ version 7.2 (Centers for Disease Control and Prevention, Atlanta, GA, USA). Les variables quantitatives sont présentées sous forme de moyennes, et les variables qualitatives sous forme de fréquences et de pourcentages. Les comparaisons de proportions ont été effectuées au moyen du test du chi-deux (X^2^). Le test exact de Fisher a été privilégié pour les paramètres d'échelle ordinale (stades pT, pN, pM) ou lorsque les effectifs étaient insuffisants. Les associations entre marqueurs et progression tumorale ont été estimées par le calcul des odds ratios (OR) avec un intervalle de confiance (IC) à 95%. Une valeur de p < 0,05 a été considérée comme statistiquement significative.

## Résultats

**Flux des patients:** entre 2016 et 2020, 112 dossiers de patients atteints d'un adénocarcinome prostatique ont été identifiés au niveau de l'Institut Pasteur du Maroc et des centres d'anatomie pathologique partenaires de Casablanca. Vingt-quatre (24) dossiers ont été exclus de l'étude: 13 pour matériel biologique insuffisant et 11 pour données cliniques incomplètes. L'analyse finale a ainsi porté sur une cohorte de 88 patients.

**Caractéristiques clinico-pathologiques de la cohorte:** sur les dossiers initialement identifiés entre 2016 et 2020, 88 patients répondaient aux critères d'inclusion et ont été analysés. L'âge des patients au moment du diagnostic variait entre 50 et 90 ans, avec une moyenne d'âge de 68 ans. Les caractéristiques cliniques initiales révèlent un toucher rectal anormal chez 77,27% des sujets (n=68). La valeur moyenne du PSA pré-thérapeutique était de 77,42 ng/ml (extrêmes: 3,68 - 1000 ng/ml), avec une majorité de patients (71,59%) présentant un taux supérieur à 10 ng/ml. Sur le plan histoprognostique, 48,87% des patients présentaient un score de Gleason élevé (≥ 8). L'analyse du stade TNM montre que 37,50% des cas étaient à un stade localement avancé (T3). Une atteinte ganglionnaire régionale (pN+) a été documentée chez 28,4% des patients, tandis que 13,64% présentaient des métastases à distance (M+) au moment de l'inclusion. Les données détaillées sont présentées dans le Tableau 1.

**Tableau 1:** caractéristiques clinico-pathologiques des patients atteints d'adénocarcinome prostatique (N = 88)

Paramètres clinico-pathologiques	Effectif (n)	Fréquence (%)
**Âge (ans)**		
≤ 60	40	45,5
> 60	48	54,5
**Toucher rectal**		
Normal	20	22,7
Anormal	68	77,3
**PSA initial (ng/ml)**		
≤ 10	25	28,4
> 10	63	71,6
**Score de Gleason**		
Gleason 6 (3+3)	17	19,3
Gleason 7 (3+4)	28	31,8
Gleason 8 (4+4)	15	17,1
Gleason 9 (4+5)	28	31,8
**Stade clinique (T)**		
T2 (Organe-confiné)	55	62,5
T3 (Stade avancé)	33	37,5
**Atteinte ganglionnaire (pN)**		
N0 (Absente)	63	71,6
N+ (Présente)	25	28,4
**Métastases (pM)**		
M0 (Absentes)	76	86,4
M+ (Présentes)	12	13,6

PSA: antigène spécifique de la prostate; TNM: tumor, node, metastasis; pN: invasion ganglionnaire locorégionale; pM: métastases à distance

**Expression du récepteur aux androgènes (RA) et corrélations clinico-pathologiques:** parmi les 88 cas étudiés, une forte expression du RA a été observée chez 67 patients (76,13%), tandis qu'une faible expression a été notée dans 21 cas (23,86%). Une différence statistiquement significative d'expression du RA a été constatée entre les deux groupes (p < 0,0001): les cancers résistants à la castration (CPRC) présentaient une forte expression dans 54 cas (80,59%) contre seulement 13 cas (19,40%) pour les cancers hormono-sensibles (HS). La surexpression du RA était significativement associée à un score de Gleason élevé (≥ 7) chez les patients résistants à la castration (OR: 44,00; IC 95%: 5,24-368,78; p = 0,0005). Dans le groupe HS, cette surexpression n'a été observée que dans les cas présentant un score de Gleason élevé. Concernant le taux de PSA, une corrélation majeure avec la surexpression du RA a été identifiée chez les patients CPRC avec un taux de PSA > 10 ng/ml (96,15%; p = 0,0001).

Une association significative a également été établie entre la surexpression du RA, l'âge > 60 ans (p = 0,0049) et le degré d'invasion ganglionnaire loco-régionale (pN+) (p = 0,0270). En revanche, aucune corrélation statistiquement significative n'a été mise en évidence avec le stade TNM ou la présence de métastases à distance (pM) (Tableau 2).

**Tableau 2:** corrélation entre la surexpression protéique du RA et les caractéristiques clinico-pathologiques (N = 88)

Paramètres clinico-pathologiques	p-value	Odds Ratio (IC 95%)
**Score de Gleason (≥ 7)**	0,0005	44,00 (5,24 - 368,78)
**Type de cancer (CPRC vs HS)**	< 0,0001	16,61 (4,75 - 58,10)
**Taux de PSA (> 10 ng/ml)**	0,0001	43,75 (6,72 - 284,61)
**Âge (> 60 ans)**	0,0049	11,66 (2,10 - 64,55)
**Invasion ganglionnaire (pN+)**	0,0270	10,66 (1,30 - 86,93)
**Stade TNM (T3 vs T2)**	0,4782	0,33 (0,01 - 6,94)

RA: récepteur aux androgènes; CPRC: cancer de la prostate résistant à la castration; HS: hormono-sensible; pN+: invasion ganglionnaire locorégionale ; IC: intervalle de confiance

**Figure 1:**
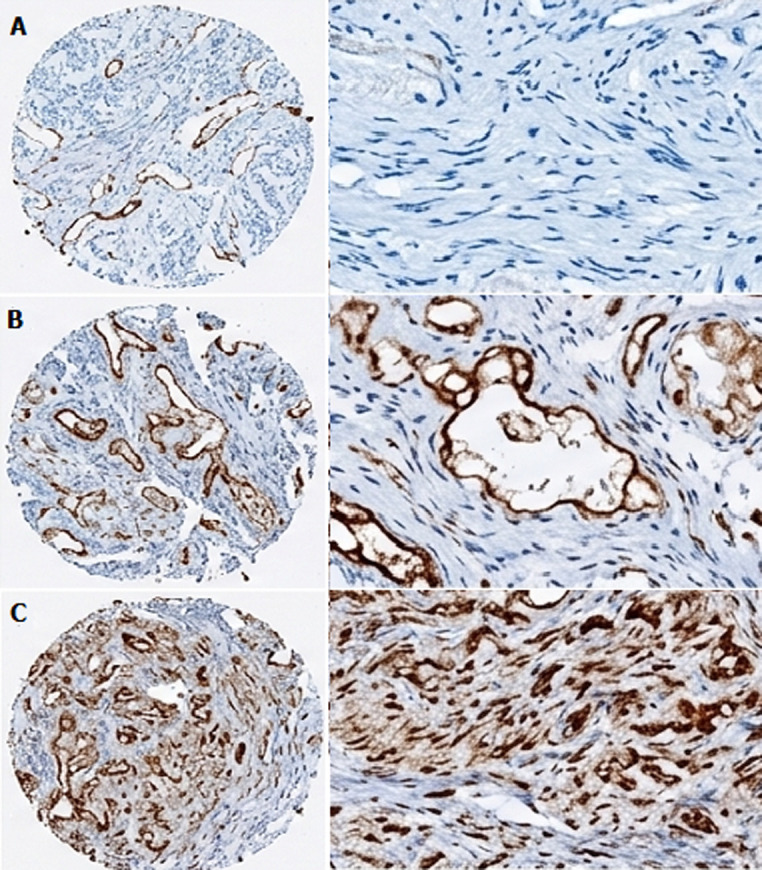
caractéristiques de l'immuno-marquage de la sous-unité NF-κB/p65 dans l'adénocarcinome prostatique: A) faible immunomarquage cytoplasmique associé à une absence de marquage nucléaire (grossissement x200); B) marquage cytoplasmique intense avec une faible translocation nucléaire (grossissement x200); C) marquage intense à la fois cytoplasmique et nucléaire, indiquant une activation du facteur de transcription (grossissement x200)

**Localisation et expression des sous-unités NF-κB (p65 et p50):** la sous-unité p65 (RelA): sur l'ensemble de la cohorte (N=88), une surexpression nucléaire de p65 a été observée dans 49 cas (55,68%). Une différence significative d'expression a été notée selon le type de cancer: 39 cas de CPRC (79,59%) présentaient une forte expression nucléaire contre une faible prévalence dans le groupe HS (p = 0,0031) (Figure 1). Dans le groupe CPRC, l'expression nucléaire de p65 était significativement corrélée au stade T3 (OR: 11,08; p = 0,0064), à l'âge (OR: 5,62; p = 0,0227), au score de Gleason ≥ 7 (OR: 4,64; p = 0,0036) et à un taux de PSA élevé (OR: 4,31; p = 0,0360). Aucune corrélation n'a été établie avec l'invasion ganglionnaire (pN+) (Tableau 3). La sous-unité p50: une forte expression nucléaire de p50 a été identifiée dans 56 cas (63,63%), dont 75% (42 cas) appartenaient au groupe CPRC. La fréquence de surexpression était significativement plus élevée chez les patients résistants à la castration par rapport aux patients hormono-sensibles (p = 0,0192). L'analyse statistique montre une corrélation entre la surexpression de p50 et un score de Gleason élevé (OR: 3,20; p = 0,0244). En revanche, contrairement à p65, l'expression de p50 n'était pas corrélée de manière significative à l'âge, au stade TNM, au taux de PSA ou à l'atteinte ganglionnaire (pN+) (Tableau 4).

**Tableau 3:** corrélation entre l'expression nucléaire de la sous-unité p65 et les caractéristiques clinico-pathologiques (N = 88)

Paramètres clinico-pathologiques	p-value	Odds Ratio (IC 95%)
**Stade TNM (T3 vs T2)**	0,0064	11,08 (1,96 - 62,49)
**Âge (> 60 ans)**	0,0227	5,62 (1,27 - 24,86)
**Score de Gleason (≥ 7)**	0,0036	4,64 (1,65 - 13,07)
**Taux de PSA (> 10 ng/ml)**	0,0360	4,31 (1,10 - 16,93)
**Type de cancer (CPRC vs HS)**	0,0031	4,10 (1,60 - 10,47)
**Invasion ganglionnaire (pN+)**	0,5699	1,66 (0,28 - 9,70)

pN+: invasion ganglionnaire locorégionale; IC: intervalle de confiance; PSA: antigène spécifique de la prostate; CPRC: cancer de la prostate résistant à la castration; HS: hormono-sensible

**Tableau 4:** corrélation entre l'expression nucléaire de la sous-unité p50 et les caractéristiques clinico-pathologiques (N = 88)

Paramètres clinico-pathologiques	p-value	Odds Ratio (IC 95%)
**Type de cancer (CPRC vs HS)**	0,0192	3,00 (1,19 - 7,52)
**Score de Gleason (≥ 7)**	0,0244	3,20 (1,16 - 8,85)
**Stade TNM (T3 vs T2)**	0,1879	2,83 (0,60 - 13,35)
**Invasion ganglionnaire (pN+)**	0,3103	2,62 (0,40 - 16,93)
**Taux de PSA (> 10 ng/ml)**	0,1810	2,50 (0,65 - 9,57)
**Âge (> 60 ans)**	0,2845	2,16 (0,52 - 8,92)

IC: intervalle de confiance; PSA: antigène spécifique de la prostate; CPRC: cancer de la prostate résistant à la castration; HS: hormono-sensible; pN+: invasion ganglionnaire locorégionale

**Expression de la sous-unité RelB (voie alternative NF-κB):** une surexpression nucléaire de RelB a été détectée dans 73 cas (82,95%). Parmi les patients CPRC, 52 cas (71,23%) étaient positifs, contre 21 cas (28,67%) dans le groupe HS. Une association significative a été établie entre l'expression de RelB et le type de cancer (OR: 3,71; p = 0,0254) ainsi qu'avec un score de Gleason élevé (OR: 4,55; p = 0,0246), spécifiquement dans le groupe CPRC. Bien qu'aucune corrélation n'ait été observée avec le stade TNM ou l'atteinte ganglionnaire, il est à noter que 100% des cas métastatiques exprimaient RelB. Aucune corrélation n'a été trouvée avec l'âge ou le taux de PSA (Tableau 5).

**Tableau 5:** corrélation entre l'expression nucléaire de RelB et les caractéristiques clinico-pathologiques (N = 88)

Paramètres clinico-pathologiques	p-value	Odds Ratio (IC 95%)
**Score de Gleason (≥ 7)**	0,0246	4,55 (1,21 - 17,08)
**Type de cancer (CPRC vs HS)**	0,0254	3,71 (1,17 - 11,73)
**Taux de PSA (> 10 ng/ml)**	0,1264	3,52 (0,70 - 17,73)
**Stade TNM (T3 vs T2)**	0,7638	0,75 (0,12 - 4,70)
**Atteinte ganglionnaire (pN+)**	0,7392	1,40 (0,19 - 10,14)
**Âge (> 60 ans)**	0,5882	2,00 (0,16 - 2,58)

pN+: invasion ganglionnaire locorégionale; IC: intervalle de confiance; PSA: antigène spécifique de la prostate; CPRC: cancer de la prostate résistant à la castration; HS: hormono-sensible

**Figure 2:**
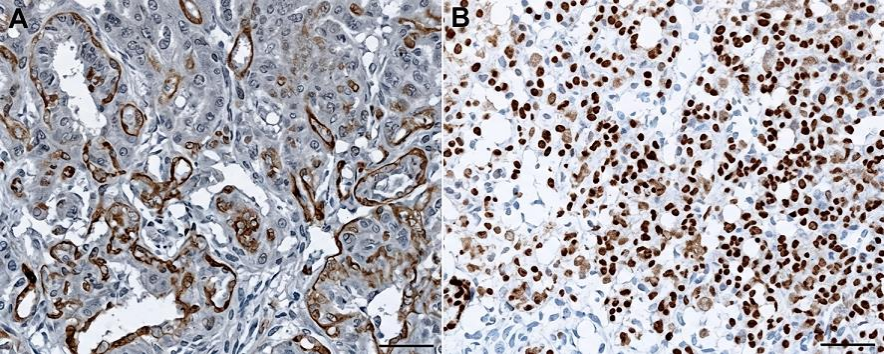
localisation subcellulaire de HER3 dans les tissus cancéreux prostatiques (grossissement x400); A) adénocarcinome prostatique hormono-sensible (HS) présentant un immunomarquage nucléaire modéré de HER3; B) adénocarcinome prostatique résistant à la castration (CPRC) montrant un marquage nucléaire intense de HER3, suggérant son implication dans la progression tumorale agressive

**Expression des récepteurs de la famille EGF (EGFR et HER3):** localisation de l'EGFR: une surexpression de l'EGFR a été identifiée dans 43 cas (48,86%). La majorité de ces cas (83,72%, n=36) appartenaient au groupe CPRC, confirmant une association forte avec la résistance à la castration (OR: 5,37; p = 0,0010). Chez les patients CPRC, la surexpression de l'EGFR était significativement corrélée au score de Gleason (OR: 6,66; p = 0,0007) et au stade TNM avancé (OR: 7,23; p = 0,0005). Aucune association significative n'a été notée avec le PSA, l'âge ou l'atteinte pN+ (Tableau 6). Localisation de HER3: une surexpression de HER3 a été observée dans 53 cas (60,22%), avec une prédominance marquée dans le groupe CPRC (79,24%, n=42) (Figure 2). Les scores élevés de HER3 étaient significativement associés au score de Gleason élevé chez les patients résistants à la castration. À l'instar de RelB, tous les cas présentant des métastases à distance étaient positifs pour HER3. L'analyse n'a pas révélé de corrélation avec l'âge, le PSA ou le stade TNM (Tableau 7).

**Tableau 6:** corrélation entre l'expression de l'EGFR et les caractéristiques clinico-pathologiques (N = 88)

Paramètres clinico-pathologiques	p-value	Odds Ratio (IC 95%)
**Score de Gleason (≥ 7)**	0,0007	6,66 (2,23 - 19,86)
**Stade TNM (T3 vs T2)**	0,0005	7,23 (2,34 - 22,31)
**Type de cancer (CPRC vs HS)**	0,0010	5,37 (1,98 - 14,59)
**Âge (> 60 ans)**	0,0513	4,48 (0,99 - 20,30)
**Atteinte ganglionnaire (pN+)**	0,1278	5,33 (0,61 - 45,99)
**Taux de PSA (> 10 ng/ml)**	0,9120	1,07 (0,27 - 4,18)

pN+: invasion ganglionnaire locorégionale; IC: intervalle de confiance; PSA: antigène spécifique de la prostate; CPRC: cancer de la prostate résistant à la castration; HS: hormono-sensible

**Tableau 7:** corrélation entre l'expression de HER3 et les caractéristiques clinico-pathologiques (N = 88)

Paramètres clinico-pathologiques	p-value	Odds Ratio (IC 95%)
**Score de Gleason (≥ 7)**	0,0012	5,12 (1,85 - 14,20)
**Type de cancer (CPRC vs HS)**	0,0028	4,25 (1,60 - 11,30)
**Stade TNM (T3 vs T2)**	0,1540	2,10 (0,75 - 5,88)
**Métastases (M+ vs M0)**	< 0,001*	Infini (100% positifs)
**Taux de PSA (> 10 ng/ml)**	0,4210	1,45 (0,52 - 4,05)
**Âge (> 60 ans)**	0,6820	1,22 (0,48 - 3,10)

* Note: Tous les cas métastatiques (100%) ayant montré une surexpression, le calcul de l'OR tend vers l'infini, confirmant une association majeure

## Discussion

Cette étude avait pour objectif d'évaluer le profil d'expression du RA, de NF-κB, de l'EGFR et de HER3 au sein d'une cohorte de 88 patients marocains. Nos principaux résultats mettent en évidence une surexpression significative de ces marqueurs dans le groupe CPRC (n=58), ainsi qu'une corrélation étroite entre le score de Gleason élevé et l'activation des voies du RA et de NF-κB. Ces données confirment que ces protéines constituent des indicateurs majeurs de la progression tumorale et de l'agressivité de l'adénocarcinome prostatique dans notre contexte.

**Profil épidémiologique et enjeux du diagnostic au Maroc:** dans notre étude, le diagnostic de l'adénocarcinome prostatique a été posé suite à l'apparition de symptômes urinaires souvent non spécifiques. L'âge moyen de nos patients (68 ans) est en parfaite adéquation avec les données récentes du Registre des Cancers du Grand Casablanca et les tendances observées en Afrique du Nord [2,10,11]. Un toucher rectal suspect et une élévation du PSA constituent toujours les piliers du dépistage au Maroc, bien que l'on observe une transition récente vers l'utilisation accrue de l'IRM multiparamétrique [12]. Concernant le stade tumoral, 37,50% de nos patients présentaient un stade T3. L'évolution vers le cancer de la prostate résistant à la castration (CPRC) reste le défi majeur: dans notre série, 70% des patients CPRC présentaient des métastases à distance, un taux qui souligne l'agressivité de la maladie dans notre contexte, où le diagnostic est souvent plus tardif que dans les pays occidentaux [13]. Cette situation impose une application rigoureuse des dernières recommandations de l'AFU publiées fin 2024 et actuellement en vigueur pour la période 2025-2026 [14]. L'auteur a pu approfondir ces concepts de progression tumorale lors d'un stage de recherche spécialisé effectué en 2013 à l'Institut du cancer de Montréal, soulignant l'importance de la transition vers des biomarqueurs plus précis que le PSA seul.

**NF-κB: un médiateur clé de la résistance thérapeutique:** l'identification de biomarqueurs comme NF-κB est cruciale pour stratifier le risque de progression. NF-κB régule une multitude de gènes impliqués dans la survie et l'invasion tumorale [15]. Nos résultats confirment une forte expression nucléaire de la sous-unité p65 dans le CPRC (p = 0,0005), corrélée significativement au grade de Gleason. Ces données s'alignent avec les méta-analyses récentes montrant que l'activation de la voie classique de NF-κB est un moteur de la résistance aux anti-androgènes de nouvelle génération [16].

L'aspect le plus novateur de notre étude est la prévalence de la sous-unité RelB (voie alternative), détectée dans 82,95% des cas. Sa corrélation avec un score de Gleason élevé, indépendamment du statut hormonal, suggère que la voie alternative de NF-κB pourrait être activée précocement dans la progression tumorale, rejoignant les observations de Koumakpayi *et al*. sur l'hétérogénéité moléculaire du CaP [17]. Contrairement aux travaux de Diallo *et al*. [18], nous avons trouvé une corrélation entre p65 et le PSA, ce qui renforce l'idée que NF-κB influence directement la signalisation du récepteur aux androgènes (RA) [19,20]. Cette voie est désormais reconnue pour son rôle majeur dans la résistance aux immunothérapies, suite aux preuves solides apportées par les travaux de Yu *et al*. publiés fin 2025 [21]. Ces résultats complètent les données historiques sur les métastases ganglionnaires [22,23] et la récidive biochimique liée à l'activation de NF-κB [24].

**L'Axe HER3/NF-κB: vers de nouvelles cibles thérapeutiques:** une contribution majeure de notre travail est la mise en évidence de l'interaction entre les récepteurs de la famille EGF et le RA. Nous avons observé que la quasi-totalité des patients exprimant l'EGFR (35/36) surexprimait également le RA, confirmant l'existence d'un crosstalk moléculaire favorisant la survie cellulaire sous privation androgénique [25-27]. Désormais, l'axe HER3 s'impose comme une cible thérapeutique de premier plan, porté par l'émergence des conjugués anticorps-médicaments (ADC) ciblant spécifiquement ce récepteur. Les résultats récents des essais cliniques sur le Patritumab Deruxtecan (HER3-DXd) confirment que le ciblage de HER3 permet de contourner les mécanismes de résistance aux anti-androgènes de nouvelle génération [28].

L'innovation porte sur la localisation nucléaire de HER3. Dans notre cohorte, la présence nucléaire d'HER3 est fortement corrélée à la résistance à la castration et au grade de Gleason élevé [29,30]. Nos résultats soutiennent l'hypothèse selon laquelle HER3 nucléaire pourrait agir comme un co-activateur de NF-κB, facilitant la progression vers des stades métastatiques [31]. Cette observation est capitale: 100% de nos cas métastatiques étaient HER3+, positionnant ce récepteur comme un biomarqueur pronostique de premier plan pour la population marocaine [32]. Bien que cette étude soit centrée sur une cohorte marocaine, la mise en évidence de l'axe HER3/NF-κB comme moteur de la résistance à la castration apporte des données précieuses pour les populations d'Afrique du Nord et de la région MENA (Middle East & North Africa). Ces résultats suggèrent que les caractéristiques moléculaires observées ici pourraient servir de base à des études multicentriques internationales visant à valider ces biomarqueurs dans des contextes épidémiologiques similaires.

Malgré la pertinence de nos résultats, cette étude présente certaines limites. Son caractère rétrospectif a pu induire des biais de sélection liés aux dossiers incomplets, bien que ce risque ait été atténué par un échantillonnage exhaustif. De plus, bien que notre cohorte soit multicentrique à l'échelle de Casablanca (N=88), une extension à d'autres régions du Maroc permettrait d'accroître la représentativité nationale de nos données. Enfin, l'absence de données de survie à long terme constitue une limite importante, restreignant l'interprétation de la valeur pronostique de NF-κB et HER3 sur la survie globale et la survie sans progression. La généralisabilité de nos résultats est soutenue par le caractère multicentrique du recrutement au sein de la plus grande métropole du Maroc, regroupant une diversité de profils socio-démographiques. L'utilisation de la technologie TMA et la standardisation des protocoles immunohistochimiques à l'Institut Pasteur assurent une reproductibilité technique élevée. Néanmoins, la validité externe de cette étude pourrait être influencée par les spécificités génétiques et environnementales de la population locale. Bien que nos corrélations entre HER3, NF-κB et l'agressivité tumorale soient cohérentes avec les données de la littérature internationale, une validation sur des cohortes issues d'autres régions du Royaume est nécessaire pour confirmer la représentativité nationale de ce profil moléculaire.

En conséquence, une interprétation prudente s'impose: ces biomarqueurs doivent être considérés pour l'instant comme des indicateurs d'agressivité tumorale plutôt que comme des facteurs pronostiques validés, ouvrant toutefois la voie à de futures investigations sur le ciblage thérapeutique du CPRC au Maroc.

## Conclusion

Notre étude souligne l'importance des altérations moléculaires dans la progression de l'adénocarcinome prostatique vers la résistance à la castration au sein d'une cohorte marocaine. Nos résultats démontrent que la translocation nucléaire des sous-unités de NF-κB (p65, p50 et RelB), ainsi que la surexpression de l'EGFR et de HER3, sont étroitement corrélées à l'agressivité tumorale et au score de Gleason. L'apport majeur de ce travail réside dans l'identification de la localisation nucléaire de HER3 comme un marqueur potentiel de l'échappement hormonal et de l'invasion métastatique. L'interaction suggérée entre la voie de signalisation alternative de NF-κB et les récepteurs de la famille EGF ouvre des perspectives prometteuses pour l'identification de nouvelles cibles thérapeutiques. À l'ère de la médecine de précision en Afrique, l'intégration de ces biomarqueurs dans la pratique clinique pourrait permettre une meilleure stratification des patients à haut risque et optimiser la prise en charge du cancer de la prostate au Maroc.

### Etat des connaissances sur le sujet

- Le cancer de la prostate (CaP) évolue fréquemment vers un stade de résistance à la castration (CPRC), où la signalisation du récepteur aux androgènes (RA) est réactivée par des mécanismes moléculaires complexes;
- Le facteur de transcription NF-κB est reconnu comme un régulateur majeur de la survie cellulaire, mais son interaction avec les récepteurs à l'EGF dans la résistance thérapeutique reste peu caractérisée cliniquement en Afrique du Nord;
- La localisation nucléaire des sous-unités de NF-κB est souvent associée à l'agressivité tumorale, bien que les données sur la sous-unité RelB (voie alternative) soient encore limitées dans les cohortes de patients.

### Contribution de notre étude à la connaissance

- Cette étude apporte une caractérisation inédite de l'expression des trois sous-unités de NF-κB (p65, p50 et RelB) chez des patients marocains, confirmant leur rôle clé dans l'évolution vers l'hormono-indépendance;
- Elle démontre une corrélation significative entre la surexpression nucléaire de p65 et de RelB et des scores de Gleason élevés et des stades tumoraux avancés (T3) dans la population étudiée;
- L'originalité majeure de ce travail réside dans la mise en évidence de la localisation nucléaire de HER3 comme biomarqueur présent dans 100% des cas métastatiques de la cohorte, et ces résultats suggèrent que l'axe HER3/NF-κB constitue une cible pronostique et thérapeutique prometteuse pour améliorer la prise en charge personnalisée du CaP au Maroc.

## Remerciements

Les auteurs expriment leur gratitude aux docteurs Fred Saad et Anne-Marie Mes-Masson pour l'accueil au sein de leur laboratoire à l'Institut du cancer de Montréal en 2013. L'initiation aux concepts de biomarqueurs pronostiques du cancer de la prostate lors de ce stage initial a permis de jeter les bases de ce travail. Les auteurs remercient chaleureusement le personnel du service d'Anatomie et Cytologie Pathologiques de l'Institut Pasteur de Casablanca pour leur son expertise technique et son soutien précieux dans la réalisation des analyses immunohistochimiques. Enfin, les auteurs tiennent à remercier l'ensemble des urologues du secteur privé ainsi que ceux du CHU de Casablanca pour leur contribution essentielle à la collecte des échantillons cliniques et des données patients, indispensable à l'aboutissement de cette étude.
